# Succesful treatment of diffuse sclerosing osteomyelitis of the mandible/mandibular chronic non-bacterial osteitis with intravenous pamidronate: resolution of pain and radiographic bone inflammation with improved cosmetic appearance - a case study

**DOI:** 10.1186/1546-0096-12-S1-P141

**Published:** 2014-09-17

**Authors:** Paivi Miettunen, Alexandra Rice, Xing Chang Wei, Weiming Yu

**Affiliations:** 1 Pediatrics, University of Calgary, Calgary, Canada; 2 Diagnostic Imaging, University of Calgary, Calgary, Canada; 3 Pathology, University of Calgary, Calgary, Canada

## Introduction

Diffuse sclerosing osteomyelitis of the manidble ( DSMO) is a form of chronic non-bacterial osteitis (CNO) and predominantly affects patients < 18 years. It can result in cosmetic disfigurement. No uniformly effective treatment exists. Intravenous pamidronate (IV-PAM) has been reported to be effective in multifocal CNO.

## Objectives

To describe clinical and radiologic outcome of a DSMO patient following treatment with IV-PAM.

## Methods

A 4-year old Caucasian female was diagnosed with DSMO and prospectively followed from 2007-2014. She presented with a 20-month history of facial asymmetry and painful bony expansion of the left hemimandible. CT-scan revealed bone expansion suggestive of fibrous dysplasia, but two consecutive bone biopsies revealed inflammatory cells only. Infectious osteomyeltis was also suspected, but cultures were negative and IV and oral antibiotics were of no benefit. Focal MRI revealed bone marrow and soft tissue edema with periosteal reaction, consistent with DSMO. Subsequent whole-body MRI revealed no other bony lesions. Naproxen was of no benefit and she was started on monthly 1-day IV-PAM infusions (1st dose 0.5mg/kg; each subsequent dose: 1mg/kg). The response to treatment was assessed according to visual analogue score for pain ( VAS, 0=no pain; 10=maximum pain), sequential MRIs and clinical photos.

## Results

She received 8 monthly IV-PAM infusions. After 1st dose, VAS decreased from 10 to 0. MRI documented resolution of abnormal signal at 5 months with gradual mandibular remodelling. Clinical photos confirmed resolution of facial asymmeter over 5 years with sustained improvement at 7 years. She had a minor MRI confirmed flare at 67 months which responded to Naproxen monotherapy. She remains asymptomatic at 84 month follow-up.

## Conclusion

DSMO is challenging to diagnose and treat due to its rareness and lack of uniformly effective treatment. IV-PAM was effective in this young refractory patient and resulted in resolution of pain, mandibular remodelling and cosmetic improvement.

## Disclosure of interest

P. Miettunen: grant / research support from: Novartis. A. Rice: none declared. X. C. Wei: none declared. W. Yu: none declared.

